# E-Predict: a computational strategy for species identification based on observed DNA microarray hybridization patterns

**DOI:** 10.1186/gb-2005-6-9-r78

**Published:** 2005-08-30

**Authors:** Anatoly Urisman, Kael F Fischer, Charles Y Chiu, Amy L Kistler, Shoshannah Beck, David Wang, Joseph L DeRisi

**Affiliations:** 1 Department of Biochemistry and Biophysics, University of California San Francisco, San Francisco, CA 94143, USA; 2 Biomedical Sciences Graduate Program, University of California San Francisco, San Francisco, CA 94143, USA; 3 Department of Infectious Diseases, University of California San Francisco, San Francisco, CA 94143, USA; 4 Departments of Molecular Microbiology and Pathology and Immunology, Washington University School of Medicine, Saint Louis, MO 63110, USA

## Abstract

DNA microarrays may be used to identify microbial species present in environmental and clinical samples. However, automated tools for reliable species identification based on observed microarray hybridization patterns are lacking. We present an algorithm, E-Predict, for microarray-based species identification. E-Predict compares observed hybridization patterns with theoretical energy profiles representing different species. We demonstrate the application of the algorithm to viral detection in a set of clinical samples and discuss its relevance to other metagenomic applications.

## Abstract 2

An algorithm, E-Predict, for microarray-based species identification is presented. E-Predict compares an observed hybridization pattern with a set of theoretical energy profiles. Each profile represents a species that may be identified.

## Background

Metagenomics, an emerging field of biology, utilizes DNA sequence data to study unculturable microorganisms found in the natural environment. Metagenomic applications include studies of diversity and ecology in microbial communities, detection and identification of representative species in environmental and clinically relevant samples, and discovery of genes or organisms with novel or useful functional properties (for recent reviews, see [1-4]).

Common to all of these applications is the task of identifying (and often quantifying the abundance of) individual genes, species, or even groups of species from the large and often complex sequence space being explored. In the most general approach, shotgun sequencing is used to both identify and quantify individual sequences in a sample of interest [5-8]. In a more targeted approach, polymerase chain reaction (PCR) is used to amplify a particular subset of sequences, which can then be cloned and analyzed. For example, 16S rRNA sequences are frequently used to identify bacterial and archaeal species [9-12]. Another approach is based on functional screening of shotgun expression libraries to identify DNA fragments that encode proteins with desirable activities [13-15].

DNA microarrays are also emerging as an important tool in metagenomics [2,16-18]. Particularly in applications concerned with real-time identification of known or related species, microarrays provide a practical high-throughput alternative to costly and time-consuming cloning and repetitive sequencing. For example, as previously reported, DNA microarrays have successfully been used to detect known viruses [19-22] and to discover a novel human viral pathogen [23]. Other metagenomic applications in which microarrays have great potential include monitoring food and water quality [24], tracking bioremediation progress [2,25], and assessment of biologic threat [26].

Use of DNA microarrays in metagenomics introduces a series of analytical challenges. First, the sequence space to explore may be very large, especially in the case of environmental samples. Given the technologic constraints on the total number of probes that can be placed on a microarray, improved algorithms are required for optimal probe selection to maximize coverage. Second, microarray data generated in metagenomic studies can be very complex. In the case of viral diagnostics, nucleic acid extracted from clinical specimens usually contains host and bacterial contaminants in addition to viral RNA and DNA. As a result, hybridization patterns are complicated by substantial amounts of noise introduced by specific and nonspecific cross-hybridization that cannot be anticipated or controlled. Third, multiple and potentially closely related species may be present in a single sample, resulting in complex or even overlapping hybridization patterns. Finally, a species identification strategy based on the use of experimentally derived patterns alone is not feasible, because such empirical controls can be obtained only for a limited number of species available as pure cultures or genomic clones. New analytical tools capable of overcoming these challenges are acutely needed.

We have previously reported the development of a DNA microarray-based platform for viral detection and discovery [23] (NCBI GEO [27], accession GPL366). Briefly, the platform employs a spotted 70-mer oligonucleotide microarray containing approximately 11,000 oligonucleotides, which represent the most conserved sequences from 954 distinct viruses corresponding to every NCBI reference viral genome available at the time of design. Nucleic acids are extracted from a sample of interest, typically a clinical specimen, and are amplified and labeled using random-primed reverse transcription, second strand synthesis, and PCR. The labeled DNA is then hybridized to the microarray, and hybridization patterns are analyzed to identify particular viruses that are present in the sample.

**Figure 1:**
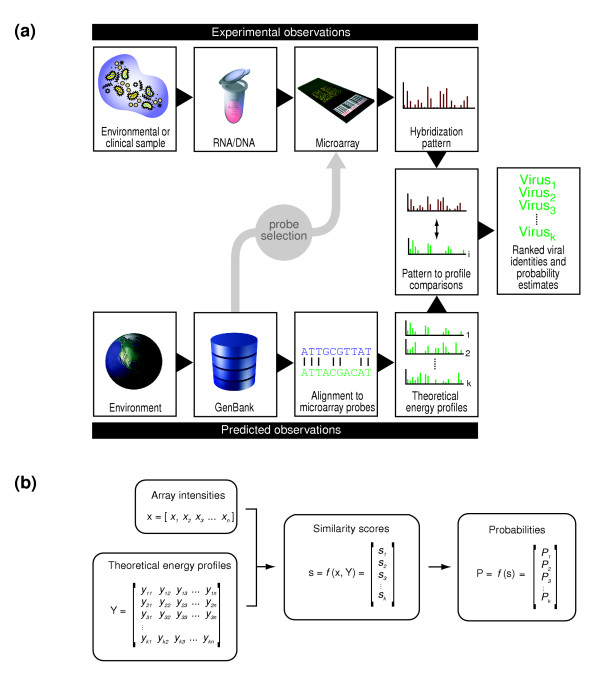
E-Predict algorithm. **(a) **Nucleic acid from an environmental or clinical sample is labeled and hybridized to a species detection microarray. The resulting hybridization pattern is compared with a set of theoretical hybridization energy profiles computed for every species of interest. Energy profiles attaining statistically significant comparison scores suggest the presence of the corresponding species in the sample. **(b) **Observed hybridization intensities are represented by a row vector x, where each intensity value corresponds to an oligonucleotide on the microarray. Theoretical hybridization energy profiles form a matrix of energy values, Y, where each row represents a profile, and each column corresponds to an oligonucleotide in x. A suitable similarity metric function compares x with each row of Y to produce a column vector of similarity scores, s. Statistical significance of the individual scores in s is estimated to produce the output column vector of probabilities, P, where each probability value corresponds to a profile in Y.

Here we report a computational strategy, called E-Predict, for species identification based on observed microarray hybridization patterns (Figure 1a). Using this strategy, an observed pattern of intensities is compared with a set of theoretical hybridization energy profiles, representing species with known genomic sequence. We illustrate the use of E-Predict on data obtained with our viral detection microarray and demonstrate its effectiveness in identifying viral species in a variety of clinical specimens. Based on these results, we argue that E-Predict is relevant for a broad range of microarray-based metagenomic applications.

## Results

### The E-Predict algorithm

Theoretical hybridization energy profiles were computed for every completely sequenced reference viral genome available in GenBank as of July 2004 (1,229 distinct viruses). This set of profiles included all viruses represented on the microarray and many viruses whose genomes became available after the array design had been completed. All microarray oligonucleotides expected to hybridize to a given viral genome were identified using nucleotide BLAST (basic local alignment search tool) alignment [28]. Free energy of hybridization (ΔG) was then computed for each alignment using the nearest neighbor method [29,30]. Oligonucleotides that failed to produce a BLAST alignment were assumed to have hybridization energies equal to zero. Thus, a given theoretical energy profile consists of the non-zero hybridization energies calculated for the subset of oligonucleotides producing a BLAST alignment to the corresponding genome. Collectively, the energy profiles of all the viruses constitute a sparsely populated energy matrix, in which each row corresponds to a viral species and each column corresponds to an oligonucleotide from the microarray (Figure 1b).

The general E-Predict algorithm for interpreting observed hybridization patterns is shown in Figure 1b. A vector of oligonucleotide intensities is normalized and compared with every normalized profile in the energy matrix using a simple similarity metric, resulting in a vector of raw similarity scores. Each element in this vector denotes the similarity between the observed pattern and one of the predicted profiles for a species represented in the energy matrix. The statistical significance of the raw similarity scores is estimated using a set of experimentally obtained null probability distributions. Profiles associated with statistically significant similarity scores suggest the presence of the corresponding viral species in the sample.

### Normalization and similarity metric choice

In order to optimize the ability of E-Predict to discriminate between true positive and true negative predictions, we first evaluated the performance of several commonly used normalizations and similarity metrics. For this purpose we constructed a training dataset of 32 microarrays obtained from samples known to be infected by specific viruses. Fifteen microarrays represented independent hybridizations of RNA extracted from HeLa cells - a human cell line that is permanently infected with human papillomavirus (HPV) type 18. The remaining microarrays were obtained from 17 independent clinical specimens from children with respiratory tract infections. Ten specimens contained respiratory syncytial virus (RSV) and seven contained influenza A virus (FluA), as determined by direct fluorescent antibody (DFA) test.

**Figure 2:**
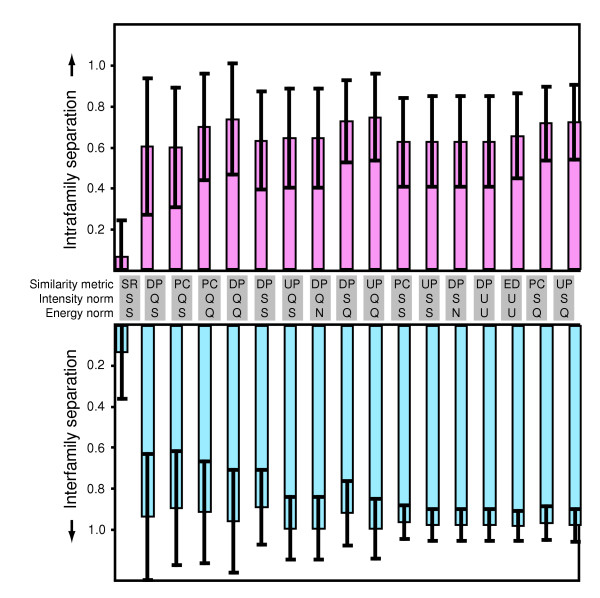
Evaluation of normalization and similarity metric parameters. A training set of 32 microarrays was used to evaluate all nonequivalent combinations of intensity and energy vector normalization (N, none; Q, quadratic; S, sum; U, unit-vector) and similarity metric (DP, dot product; ED, similarity based on Euclidean distance; PC, Pearson correlation; SR, Spearman rank correlation; UP, uncentered Pearson correlation) parameters. For each combination of parameters, intrafamily and interfamily separations were calculated for each microarray as the score of the virus profile matching the virus present in the sample minus the score of the best scoring nonmatch profile from the same or a different virus family (top and bottom panels, respectively), normalized by the range of all scores on that microarray. Bars represent the mean, and error bars represent the standard deviation (±) of separation values from all microarrays. The best performing combinations are shown in order of increasing performance (calculated as the product of the intrafamily and interfamily separation means divided by the corresponding standard deviations).

Intensity and energy vectors were independently normalized using sum, quadratic, unit-vector, or no normalization (Table 1). Similarity scores between the vectors were computed using dot product, Pearson correlation, uncentered Pearson correlation, Spearman rank correlation, or similarity based on Euclidean distance (Table 2). All nonequivalent combinations of intensity vector normalization, energy vector normalization, and similarity metrics were evaluated. For each combination, similarity scores were obtained by comparing every microarray in the training dataset with every virus profile in the energy matrix. The performance of each combination was then evaluated by calculating the separation between the score obtained for the correct (match) virus profile and the best scoring nonmatch profile from either the same or a different virus family (Figure 2a and Figure 2b, respectively). We defined separation as the difference between the similarity scores of a match and the appropriate nonmatch profiles, divided by the range of all similarity scores on a given microarray. Using this statistic, a value of one corresponds to the best possible separation, a value of zero corresponds to no separation, and negative values represent cases in which a match profile is assigned a score lower than a nonmatch profile.

With the exception of Spearman rank correlation, all considered metrics assigned the highest similarity scores to the match profiles on all 32 microarrays, independent of normalization choice. Not surprisingly, separation between interfamily profiles was greater than that between intrafamily profiles. In addition, changes in normalization and similarity metric had greater impact on intrafamily than on interfamily separation. The best overall separation was determined by calculating the product of the means of the intrafamily and interfamily separations divided by the corresponding standard deviations. Sum normalization of the intensity vectors, quadratic normalization of the energy vectors, and uncentered Pearson correlation as the similarity metric achieved the highest overall separation, producing a mean intrafamily separation of 0.69 (standard deviation 0.17) and a mean interfamily separation of 0.93 (standard deviation 0.08). Therefore, we settled on this combination of normalization and similarity metric parameters as our method of choice.

### Significance estimation

Raw similarity scores, as described above, provide an effective means of ranking viral energy profiles based on similarity to an observed hybridization pattern. However, such ranking provides no explicit information regarding the likelihood that viruses corresponding to the best scoring profiles are actually present in a sample under investigation. For example, two profiles may have identical high scores, but one of the scores may reflect a true positive whereas the other may be the result of over-representation of cross-hybridizing oligonucleotides in a profile.

To facilitate the interpretation of individual raw similarity scores, we sought to develop a test of their statistical significance. For this purpose, we obtained empirical distributions of the scores for every virus profile in the energy matrix. The distributions were based on 1,009 independent microarray experiments collected from a wide range of clinical and nonclinical samples representing different tissues, cell types, and nucleic acid complexities. Given such sample diversity, we assumed that any given virus was present in only a small fraction of all samples. Therefore, the empirical distributions are essentially distributions of true negative scores. The log_e_-transformed similarity scores were approximately normally distributed. Outliers on the right tails of the distributions, assumed to be true positives, were removed (see Materials and methods, below), and parameters of the null distributions were estimated as the mean and standard deviation of the remaining observations. These parameters were used to calculate the probability associated with any observed similarity score. Probabilities obtained this way should be interpreted as one-tail *P *values for the null hypothesis, that the virus represented by the profile is not present in the sample.

**Figure 3:**
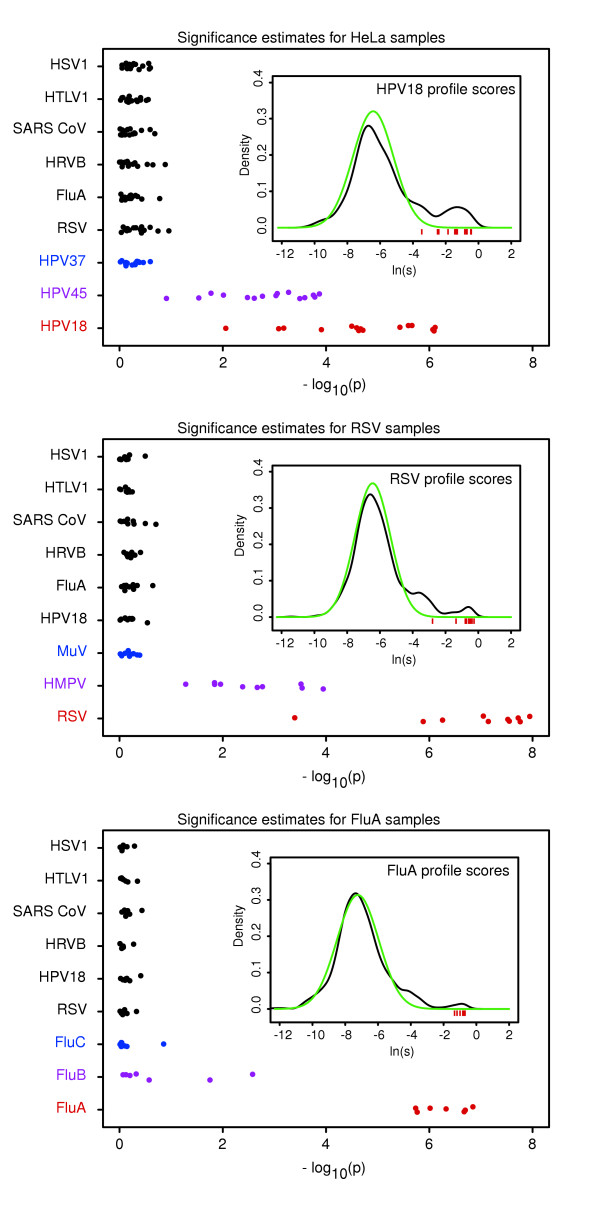
Estimation of significance of individual similarity scores. Probabilities associated with the similarity scores of nine representative virus profiles obtained for the 15 HeLa, 10 respiratory syncytial virus (RSV), and seven influenza A virus (FluA) microarrays from the training dataset are shown in the top, center, and bottom panels, respectively. Each circle represents one microarray, and vertical 'jitter' is used to resolve individual circles. Probabilities for virus profiles from seven diverse virus families are included with each microarray set: herpes simplex virus (HSV)1; human T-lymphotropic virus (HTLV)1; severe acute respiratory syndrome coronavirus (SARS CoV); human rhinovirus B (HRV)B; FluA; human RSV; and three human papillomaviruses (HPV)18. Red circles represent match and black circles nonmatch interfamily profiles. Two intrafamily nonmatch profiles are also included and are different for the three microarray sets. The most closely related intrafamily profiles are represented by purple circles: HPV45, human metapneumovirus (HMPV), and influenza B virus (FluB). More distant intrafamily profiles are shown in blue: HPV37, mumps virus (MuV), and influenza C virus (FluC). The inset in each panel shows a normalized histogram (density) of the empirical distribution of log-transformed similarity scores for a match profile (black curve) and the corresponding normal fit representing true negative scores (green curve). Inset red bars depict observed log-transformed similarity scores corresponding to the match profile probabilities (red circles).

As shown in Figure 3, the most significant similarity scores for all 32 microarrays in the training dataset were correctly matched to the virus known to be present in the input sample: HPV18 for HeLa samples, RSV for RSV-positive samples, and FluA for FluA-positive samples. Corresponding *P *values ranged between 8.7 × 10^-3 ^and 7.7 × 10^-7 ^(median 2.1 × 10^-5^), between 4.0 × 10^-4 ^and 1.4 × 10^-8 ^(median 5.1 × 10^-8^), and between 1.8 × 10^-6 ^and 1.4 × 10^-7 ^(median 4.7 × 10^-7^), respectively (Figure 3; red circles). Energy profiles of unrelated viruses from six representative families (black circles) as well as profiles of divergent members belonging to the same families as the match viruses (blue circles) had similarity scores of essentially background significance (*P *values > 0.14). Even *P *values of the most closely related intrafamily virus profiles (purple circles) were separated from those of the match viruses by more than 1.1 (HPV45), 2.1 (human metapneumovirus), and 3.4 (influenza B virus) logs. Although the *P *values obtained for these profiles are more significant than background, their similarity scores are entirely based on oligonucleotides that also belong to the match virus profiles. *P *values resulting from such profile overlaps can be easily recognized and masked if desired (see Example 3, below).

### Examples

Our laboratory is conducting a series of studies focused on human diseases suspected of having viral etiologies. The E-Predict algorithm was developed to assist in the analysis of samples obtained as part of these investigations. As an illustration of its versatility we present four example applications of E-Predict, as it is used in our laboratory.

#### Example 1

In this example, E-predict was used to interpret a hybridization pattern complicated by a low signal-to-noise ratio (Tables 3 and 4). The microarray result was obtained as part of our ongoing study of viral agents associated with acute hepatitis. Total nucleic acid from a serum sample was amplified, labeled, and hybridized to the microarray using our standard protocol (see Materials and methods, below). Despite the fact that very few oligonucleotides had intensity higher than background (Table 4), E-Predict assigned highly significant scores to hepatitis B virus (*P *= 0.002) and several closely related hepadnaviruses (Table 3). Specifically, no hepadnavirus oligonucleotide had intensity greater than 500 (for reference, background intensities are around 100, and the possible range is between 0 and 65,536). PCR with hepatitis B specific primers confirmed the presence of the virus in the sample. Complete E-Predict output for this example is available as Additional data file 1. The microarray data have been submitted to the NCBI GEO database [27] (accession GSE2228).

#### Example 2

In this example, E-Predict was used to identify the presence of two distinct viral species in the same sample (Table 5). The microarray result was obtained from a nasopharyngeal aspirate sample, which was collected as part of our ongoing investigation of childhood respiratory tract infections. On this microarray, E-Predict assigned highest significance to two unrelated viruses, namely FluA (*P *< 10^-6^) and RSV (*P *= 0.008), suggesting a double infection. The sample was independently confirmed to contain FluA and RSV, by DFA and specific PCR, respectively. Complete E-Predict output for this example is available as Additional data file 2. The microarray data have been submitted to the NCBI GEO database [27] (accession GSE2228).

#### Example 3

This example illustrates the ability of E-Predict to identify a virus that was not included in the microarray design. Table 6 shows E-Predict results for a microarray used to identify a novel coronavirus (severe acute respiratory syndrome (SARS) coronavirus (CoV)) during the 2003 outbreak of SARS, as reported previously [23,31]. Because our microarray was designed before 2003, it did not contain oligonucleotides derived from the SARS CoV genome. However, after the entire genome sequence of the virus became available [32], its theoretical energy profile was added to the E-Predict energy matrix. Reanalysis of the original SARS microarray data (NCBI GEO [27], accession GSM8528) using E-Predict revealed that the SARS CoV energy profile attained the highest similarity score and a highly significant *P *value (*P *= 1 × 10^-6^), despite the fact that the microarray, and therefore the profile, did not contain any oligonucleotides derived from the SARS CoV genome.

In addition to the SARS CoV prediction mentioned above, several astrovirus and picornavirus profiles had similarity scores with significant *P *values. However, these predictions were based on oligonucleotides corresponding to a conserved 3'-untranslated region shared by these viruses with the SARS CoV [23,33]. To identify incorrect predictions, such as these, resulting from partial profile overlaps with a match virus, we implemented an iterative version of E-Predict in which oligonucleotide intensities corresponding to the top scoring profile from one iteration are set to zero before running the next iteration. As a consequence, misleading predictions resulting from oligonucleotides shared with the top scoring profile fail to attain significant similarity scores in subsequent iterations. Conversely, only those predictions that are based on alternative oligonucleotides, namely predictions representing distinct species, remain. When iterative E-Predict was used on the SARS microarray, no astrovirus or picornavirus profile attained a statistically significant score (*P *> 0.04) in the second iteration, effectively removing these profiles from consideration. Complete E-Predict output for this example is available as Additional data file 3.

#### Example 4

**Figure 4:**
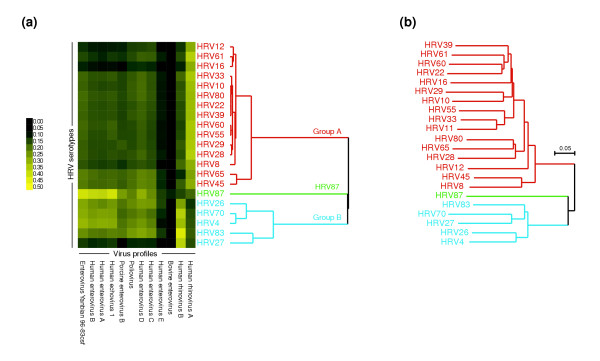
Human rhinovirus (HRV) serotype discrimination using E-Predict similarity scores. **(a) **Culture samples of 22 distinct HRV serotypes were separately hybridized to the microarray. E-Predict similarity scores were obtained for all virus profiles in the energy matrix and clustered using average linkage hierarchical clustering and Pearson correlation as the similarity metric. Virus profiles for which similarity scores could be calculated in all 22 experiments were included in the clustering. Both microarrays (rows) and virus profiles (columns) were clustered. **(b) **Published nucleotide sequences of VP1 capsid protein from the 22 HRV serotypes were aligned using ClustalX. Phylogenetic tree based on the resulting alignment is shown.

This example illustrates the use of E-Predict to discriminate between closely related viral species such as human rhinovirus (HRV) serotypes (Figure 4). Rhinoviruses are a genus in the picornavirus family, which also includes enterovirus, aphthovirus, cardiovirus, hepatovirus, and parechovirus genera. Partial sequence analysis [34-36] indicates that HRV serotypes can be divided into two major groups (A and B), with the exception of HRV87, which is more closely related to enteroviruses. Only two complete rhinovirus reference genomes are available, one for each group: HRV89 (group A) and HRV14 (group B). Energy profiles of both viruses are included in our energy profile matrix as well as profiles of several enteroviruses and other more distant members of the picornavirus family. RNA samples from cultures of 22 representative serotypes were individually hybridized to the microarray, and the results were analyzed by E-Predict. In the absence of complete genome sequence data and corresponding energy profiles for each of the 22 serotypes, the E-Predict results revealed whether a particular serotype was most similar to HRV89, HRV14, or one of the enterovirus genomes in the energy matrix. To further refine our analysis, we clustered the E-Predict similarity scores from all 22 microarrays across all picornavirus profiles (Figure 4a). The resulting cluster dendrogram of the serotypes exhibited striking similarity to a phylogenetic tree based on nucleotide sequences of VP1 capsid protein (Figure 4b; also see Ledford and coworkers [34]). Serotypes 4, 26, 27, 70, and 83 were correctly grouped together on the basis of their similarity to the profile of HRV14 (group B); HRV87 formed a separate node, and the remaining serotypes were grouped together on the basis of their similarity to the profile of HRV89 (group A). Complete E-Predict output for this example is available as Additional data file 4. The microarray data have been submitted to the NCBI GEO database [27] (accession GSE2228).

## Discussion

Identifying individual species present in a complex environmental or clinical sample is an essential component of many current and proposed metagenomic applications. Given a foundation of genomic sequence information, DNA microarrays are a high-throughput and cost-effective methodology for detecting species in an unbiased and highly parallel manner. Metagenomic applications employing DNA microarrays include characterization of microbial communities from environmental samples such as soil and water [2,17], pathogen detection in clinical specimens and field isolates [16], monitoring of bacterial contamination of food and water [24], and detection of agents involved in potential cases of bioterrorism [26].

Despite the increasing use of DNA microarrays for species detection and identification, bioinformatics tools for interpreting hybridization patterns associated with complex clinical and environmental samples are lacking. Existing methods have utilized direct visual inspection of hybridizing oligonucleotides [23,37] or inspection following clustering [19,38]. Such methods are intractable for interpreting complex hybridization patterns, are time consuming, and suffer from user bias. Improved data interpretation tools must address several challenges. First, hybridization patterns may represent signal from dozens or even hundreds of species. Also, several closely related species may be present in a sample, giving rise to overlapping hybridization signals. A likely additional source of noise is unanticipated cross-hybridization, because many of the genomes present in a complex sample may be uncharacterized. Finally, obtaining pure samples of each possible species for the purpose of generating reference hybridization patterns is impractical or impossible in most cases.

When challenged with each of these problems, E-Predict proved to be a useful tool for interpreting hybridization patterns, correctly identifying viruses from diverse viral families present in a variety of clinical samples. In particular, E-Predict does not rely on the use of empirically generated reference hybridization patterns, because species identification is based instead on theoretical hybridization energy profiles. The energy profile matrix currently represents over 1,200 distinct viruses whose complete genomic sequences are known. As new viral genomes are sequenced, profiles are added to the matrix to broaden the range of species detection. For example, addition of the SARS CoV profile enabled accurate identification of the virus, even though no oligonucleotides derived from its genome were present on the microarray. Conversely, even when a perfectly matching profile is not available because of limited sequence coverage, E-Predict will identify the closest related species, as long as such species are represented on the microarray. This feature is particularly useful for detecting novel viruses as well as for discriminating between closely related viruses such as HRV serotypes. Naturally, maximum range and precision of detection is achieved through addition of new profiles and periodic microarray updates to include specific oligonucleotides from newly sequenced species.

E-Predict is also useful in overcoming problems related to nucleic acid complexity frequently encountered in clinical samples. For example, E-Predict correctly identified hepatitis B virus in a serum sample, despite the fact that the hybridization pattern was complicated by a low signal-to-noise ratio. In another example, E-Predict deconvoluted a complex hybridization pattern, correctly suggesting the presence of two viruses (FluA and RSV) in a nasopharyngeal aspirate sample. In yet another example, iterative application of E-Predict (see Materials and methods, below) to a hybridization pattern involving oligonucleotides derived from seemingly unrelated families (coronaviridae and astroviridae) premitted objective recognition that the pattern represented the presence of only one virus (SARS CoV).

Using a training dataset of 32 microarrays derived from samples known to contain specific viral species, we identified a set of normalization and similarity metric parameters, which yielded the best discrimination between true positive and true negative species predictions. The combination of sum normalization of the intensity vectors, quadratic normalization of the energy vectors, and uncentered Pearson correlation as the similarity metric was the optimal choice for our data. However, a different set of parameters may be required for applications that use a different nucleic acid amplification or detection strategy. An independent evaluation of potentially useful normalization and similarity metric parameters is therefore recommended for each specific application of the algorithm.

Using our best combination of normalization and similarity metric parameters, we obtained a set of null distributions representing true negative scores. These distributions were based on over 1,000 independent hybridizations and the assumption that the majority of samples were negative for the presence of any given virus. Although valid for our data, this assumption will not hold for all cases. For example, in applications concerned with bacterial species detection, some species may be present in most or even all samples and others encountered only rarely. In this case, a more complicated model will be required to assess whether a specific distribution represents negative, positive, or both negative and positive scores. For example, in cases in which distributions appear bimodal, one mode may represent true negatives and the other true positives. In some cases, targeted experimental verification of a subset of representative scores may be necessary. If both positive and negative score distributions are available, then *P *values can be calculated for each distribution.

Several modifications to the algorithm may potentially result in improved prediction accuracy. First, in the current implementation oligonucleotides exhibiting nonspecific cross-hybridization are filtered and the remaining oligonucleotides are weighted equally. Because oligonucleotides exhibit a continuous range of nonspecific hybridization [20,30], a more sophisticated system of oligonucleotide weights may result in better performance. For example, using a procedure similar to that used to generate null distributions for the virus profile scores, empirical distributions can be obtained for individual oligonucleotide intensities, and individual oligonucleotide contributions may be weighted by the probabilities associated with the corresponding observed intensities. Such weighting may allow a more accurate assessment of significance.

Second, no attempt was made to normalize nucleic acid abundances of individual species, which may vary widely in different samples depending on factors such as target-to-background ratio, number of species present, and efficiency of nucleic acid extraction and amplification. Although individual nucleic acid abundances are difficult or impossible to estimate in most metagenomic applications, particularly before the corresponding species have been identified, in applications in which such estimates can be made, either experimentally or on theoretical grounds, the use of correction factors for calculating similarity scores or stratification of *P *value estimation may be needed. In addition, for highly abundant species, care should be taken to avoid saturation of individual oligonucleotides, because E-predict performance drops sharply after 20-25% of oligonucleotides in a given profile are saturated (data not shown).

Third, even though viral genomes were used as the basis for calculating energy profiles, the concept can easily be extended to other taxonomy nodes such as genera or families of viruses. This requires every sequence element to be classified at the appropriate node in the taxonomy hierarchy.

Finally, iterative use of E-Predict was intended for identification of multiple species that may be present in a sample. In this setting, it is important to distinguish between true predictions representing unique species present in the sample and misleading predictions arising from partially overlapping profiles. In each iteration it is assumed that the profile attaining the highest score corresponds to the species most likely to be among those present in the sample. When a novel species is present, this assumption may not hold because of limited oligonucleotide coverage. For instance, in the SARS CoV example, although SARS CoV attained a higher similarity score than mink astrovirus, the corresponding *P *values were comparable. However, even if mink astrovirus were the top prediction in the first iteration, SARS CoV would be the top prediction in the second iteration (*P *= 2 × 10^-6^; data not shown) and therefore would not be missed as a true positive. In our current studies *P *values in all iterations are estimated using the same set of null probability distributions. In addition, we use two iterations as our default, and essentially never need to run more than three iterations, because detection of more than two or three viruses is rare. However, iterative resolution of hundreds or thousands of species present in a sample may necessitate other normalization methods or adjustments to the null distributions for *P *value estimation. As an alternative, noniterative algorithms for analyzing overlapping profile signatures are also being explored.

In conclusion, E-Predict is a novel computational approach for species identification, which is generally applicable to a wide range of metagenomic applications using DNA microarrays. In particular, as more sequencing efforts are being directed at natural microbial communities, DNA microarrays are bound to become a central tool for various downstream applications such as identification of microbial species or detection of genes and biochemical pathways in such communities. E-Predict addresses an acute need for computational tools that are capable of interpreting the highly complex microarray data obtained through such studies. E-Predict was developed for viral species identification and therefore has immediate implications for medical diagnostics and viral discovery. In addition, the concept of theoretical energy profiles can be extended to represent other microorganisms, particular genes, or biochemical pathways.

## Materials and methods

### Sample preparation and hybridization to microarrays

All patient samples were collected according to protocols approved by the University of California San Francisco Committee on Human Research.

HeLa cells were grown to confluence in a T150 tissue culture flask in Dulbeco's modified Eagle medium supplemented with 10% fetal bovine serum and antibiotics. The cells were harvested by adding 10 ml Trizol reagent (Invitrogen, Carlsbad, CA, USA), and total RNA was isolated according to the manufacturer's protocol. A quantity of HeLa total RNA (50 ng) was used for each amplification and hybridization.

With respect to pediatric respiratory samples, frozen nasopharyngeal aspirate samples were thawed and 200 μl aliquots were used to extract RNA using RNeasy Mini Kit (Qiagen USA, Valencia, CA,USA) as follows. RLT buffer (750 μl) containing 1% 2-mercaptoethanol was added to each sample and mixed. Then, 1 ml of 100% ethanol was added, and the resulting mixture was applied to the columns in three 650 μl aliquots. The remaining steps were carried out in accordance with the manufacturer's protocol, including on-column DNase digest. RNA was eluted from the columns with 30 μl nuclease-free water, and 9 μl was used for amplification and hybridization. For the hepatitis sample, frozen serum sample was thawed and a 150 μl aliquot was used to extract total nucleic acid using MagNA Pure LC Total Nucleic Acid Isolation Kit (Roche Molecular Systems, Alameda, CA, USA), in accordance with the manufacturer's protocol. RNA was eluted in 50 μl nuclease-free water, and 9 μl was used for amplification and hybridization.

For HRV serotypes, frozen samples of low passage viral culture supernatants were thawed on ice and pre-filtered with a 0.2 μm syringe filter. Aliquots (200 μl) of the pre-filtered supernatants were treated with 600 U micrococcal nuclease (Fermentas USA, Hanover, MD, USA) in the presence of 10 mmol/l CaCl_2 _for 3 hours at 37°C. RNA was then extracted using Trizol reagent (Invitrogen), in accordance with the manufacturer's protocol. Linearized polyacrylamide (20 μg; Ambion, Austin, TX, USA) was used as the carrier during the 2-propanol precipitation. RNA was resuspended in 30 μl nuclease-free water, and 9 μl was used for amplification and hybridization.

Microarrays used in the study were essentially identical to those previously described [23]. Detailed description of the microarray platform, including oligonucleotide sequences, can be found in the NCBI GEO database [27] (accession GPL 1834). Briefly, 70-mer oligonucleotides representing the most conserved viral genomic elements were selected as 70-mers having sequence similarity (determined by nucleotide alignment) to the highest number of viral genomes [19]. Oligonucleotides were resuspended in 3 × SSC (0.45 M sodium chloride, 0.045 M sodium citrate, pH 7.0) at 50 μmol/l concentration and spotted onto poly-lysine coated glass slides [39]. Each spot on the microarray also contained a unique 'alien' sequence 70-mer (Spike70: 5'-ACC TCG CTA ACC TCT GTA TTG CTT GCC GGA CGC GAG ACA AAC CTG AAC ATT GAG AGT CAC CCT CGT TGT T-3'), spotted at a 1:50 ratio with the viral oligonucleotide to facilitate gridding of the microarrays (see below).

RNA extracted from the samples was amplified using a modified Round A-B random PCR method [40], as previously described (protocol S1 in [23]). Briefly, random-primed reverse transcription and second strand synthesis were carried out using primer A (5'-GTT TCC CAG TCA CGA TCN NNN NNN NN-3'). The resulting material was then amplified with 40 cycles of PCR using primer B (5'-GTT TCC CAG TCA CGA TC-3'). This was followed by an additional 20 cycles of PCR with primer B to incorporate aminoallyl-dUTP. The amplified material was then labeled with Cy5, and 0.1-1.0 pmol Probe70 (an oligonucleotide complementary to Spike70 containing five amino-modified bases for dye coupling: 5'-AAC AAC GAG GG[AmC6-dT] GAC TCT CAA [AmC6-dT]GT TCA GGT TTG TC[AmC6-dT] CGC GTC CGG CAA GCA A[AmC6-dT]A CAG AGG T[AmC6-dT]A GCG AGG T-3', Operon Biotechnologies, Huntsville, AL, USA) was labeled with Cy3. The Cy5 and Cy3 probes were pooled and hybridized to the microarray in 3 × SSC at 65°C overnight [39]. The Cy3 channel was used to facilitate gridding but otherwise was ignored in the data analysis. Microarrays were scanned with an Axon 4000B scanner (Axon Instruments, Union City, CA, USA) and gridded using the bundled GenePix 3.0 software.

Microarray data have been submitted to the NCBI GEO database [27] (accession GSE2228). The SARS microarray data are also available in NCBI GEO (accession GSM8528), as previously reported [23].

### Training dataset

Fifteen HeLa microarrays were chosen randomly from a set of 43 HeLa hybridizations having at least five papillomavirus oligonucleotides with sum-normalized intensities greater or equal to 0.005. Ten RSV microarrays were chosen randomly from a set of 22 clinical hybridizations having at least five paramyxovirus oligonucleotides with sum-normalized intensities greater than or equal to 0.005 and confirmed to be RSV-positive by DFA. Seven FluA microarrays were chosen from eight available clinical hybridizations having at least five orthomyxovirus oligonucleotides with sum-normalized intensities greater than or equal to 0.005 and confirmed to be FluA-positive by DFA. The eighth FluA microarray was excluded because it was also positive for RSV by visual inspection.

### Theoretical energy profiles

The energy profile matrix used in this study included all NCBI reference viral genomes (1,229) available as of July 2004 [41]. Nucleotide BLAST (blastall version 2.2.8 [42] with the default settings) was used to align microarray oligonucleotides with the viral genomes. Energies of hybridization were computed from the alignments using a program distributed with ArrayOligoSelector [30,43]. In cases in which an oligonucleotide had multiple alignments to the same genome, energy calculations were based on the highest scoring alignment. The energy profile matrix is available as Additional data file 5.

### Similarity scores

Control oligonucleotides and oligonucleotides known to result in nonspecific hybridization were removed from consideration by setting their intensities and energies to zero. The list of these oligonucleotides (Additional data file 6) was obtained by including 129 oligonucleotides with unnormalized median intensity greater than 500, calculated from 1,009 independent hybridizations described below. The list also included 137 oligonucleotides obtained by clustering of distributions of sum-normalized intensity, based on the same set of 1,009 hybridizations, and visual identification of an outlier cluster with median sum-normalized intensities significantly higher than those observed for most oligonucleotides. Energy vectors were further filtered to exclude terms with energy predictions higher than -30 kcal/mol (again by setting their values to zero), because such predictions on our platform do not correspond to detectable array intensities [30]. A profile was considered only if it had at least three oligonucleotides with non-zero energy predictions. The resulting intensity and energy vectors were normalized using appropriate normalization methods (no normalization, sum, quadratic, and unit-vector). Similarity scores were computed using an appropriate similarity metric (dot product, Pearson correlation, uncentered Pearson correlation, Spearman rank correlation, and similarity based on Euclidean distance).

### Probability estimation

Null distributions of similarity scores were obtained using a set of 1,009 hybridizations, which included all hybridizations performed on our platform to date. Similarity scores were calculated as described above using uncentered Pearson correlation as the similarity metric, and sum and quadratic normalizations for intensity and energy vectors, respectively. Scores were log-transformed. Right tail outliers corresponding to positive cases were excluded by iterative trimming of the top scores in 1% increments until the best normality fit was obtained, as judged by the Shapiro-Wilk normality test [44] (implemented in R [45]). Trimming was allowed to involve 0-25% of all scores. Over one-third of virus profiles required no trimming at all. Only a small number of profiles (34) required trimming beyond 10%, all of which corresponded to viruses frequently present in our samples. No profile required trimming of more than 17% of the scores. The resulting trimmed distributions were assumed to be normal, and their parameters were estimated as the mean and standard deviation of the included scores (Additional data file 7). Obtained parameters were used to estimate significance of individual scores as probabilities associated with observing values equal or greater than the scores. For this purpose, only profiles with at least three oligonucleotides with raw intensity greater than 100 (about two to four times background) were considered.

### Iterative E-Predict

The first iteration was carried out as described above. For each additional iteration, oligonucleotide intensities of the profile attaining the highest similarity score in the previous iteration were set to zero. The resulting intensity vector was normalized, and similarity scores and *P *values were calculated using the same normalization method, similarity metric, and null distributions as in the initial iteration.

### Clustering of human rhinovirus serotypes

Similarity scores were calculated as described above using uncentered Pearson correlation as the similarity metric, and sum and quadratic normalizations for intensity and energy vectors, respectively. Scores corresponding to picornavirus profiles were clustered using Cluster (version 2.0) [46,47] by hierarchical average linkage clustering with Pearson correlation as the similarity metric. Cluster images were obtained using Java TreeView (version 1.0.8) [48,49].

The phylogenetic tree based on nucleotide sequences of VP1 capsid protein was constructed using data from the report by Jonassen and coworkers [34]. Sequence alignment of relevant serotypes and the resulting tree were obtained using ClustalX (version 1.81 for Windows [50,51]) with default settings.

### Polymerase chain reaction

The presence of hepatitis B virus in the hepatitis sample was confirmed using primers Hep_1F (5'-GAC TCG TGG TGG ACT TCT CTC AA-3') and Hep_4R (5'-GAA AGC CCT GCG AAC CAC TGA A-3') with amplified cDNA (Round B material; see [19] for amplification details) as the template. The presence of RSV in the FluA/RSV double-infected sample was confirmed by PCR using primers AU_041 (5'-GAT GAA AAA TTA AGT GAA ATA TTA GG-3') and AU_042 (5'-GTT CAC GTA TGT TTC CAT ATT TG-3') with cDNA (Round A material; see [19] for amplification details) as the template. In both cases, amplified PCR fragments were sequenced and had at least 99% nucleotide identity to the genomes of Hepatitis B virus (GenBank: NC_003977) and RSV (GenBank: NC_001803).

### E-Predict software

The E-Predict software is available for download by any interested party [52].

## Additional data files

The following additional data are available with the online version of this paper: a text file of E-Predict output for the hepatitis example (example 1) (Additional data file 1); a text file of E-Predict output for the FluA/RSV double infection example (example 2) (Additional data file 2); a text file of E-Predict output for the SARS CoV example (example 3) (Additional data file 3); a text file of E-Predict output for the HRV serotypes example (example 4) (Additional data file 4); a tab delimited text file containing the energy profile matrix (Additional data file 5); a text file containing the list of nonspecific oligonucleotides ignored during E-Predict (Additional data file 6); a tab delimited text file containing the list of profile parameters used to estimate *P *values (Additional data file 7). A text file of E-Predict output used to evaluate normalization and similarity metric parameters (Additional data file 8).

## Supplementary Material

Additional data file 1 A text file of E-Predict output for the hepatitis example Click here for file

Additional data file 2 A text file of E-Predict output for the FluA/RSV double infection example Click here for file

Additional data file 3 A text file of E-Predict output for the SARS CoV example Click here for file

Additional data file 4 A text file of E-Predict output for the HRV serotypes example Click here for file

Additional data file 5 A tab delimited text file containing the energy profile matrix Click here for file

Additional data file 6 A text file containing the list of nonspecific oligonucleotides ignored during E-Predict Click here for file

Additional data file 7 A tab delimited text file containing the list of profile parameters used to estimate *P *values Click here for file

Additional data file 8 A text file of E-Predict output used to evaluate normalization and similarity metric parameters Click here for file

## Figures and Tables

**Table 1:** Normalization methods

Normalization	Formula	Abbreviation
None	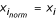	N
Sum	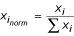	S
Quadratic	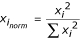	Q
Unit vector	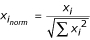	U

**Table 2:** Similarity metrics

Similarity metric	Formula	Abbreviation
Dot product	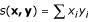	DP
Pearson correlation	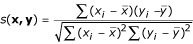	PC
Uncentered Pearson correlation	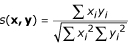	UP
Spearman rank correlation	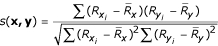	SR
Similarity based on Euclidean distance	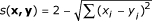	ED

**Table 3:** Example 1: Hepatitis microarray - predicted virus profiles

Taxonomy ID	Virus profile	Virus family	Similarity score	Probability
10407	Hepatitis B virus	Hepadnaviridae	0.145209	0.002451*
113194	Orangutan hepadnavirus	Hepadnaviridae	0.143754	0.002482*
68416	Woolly monkey hepatitis B virus	Hepadnaviridae	0.123794	0.003111*
35269	Woodchuck hepatitis B virus	Hepadnaviridae	0.106576	0.002896*
41952	Arctic ground squirrel hepatitis B virus	Hepadnaviridae	0.098908	0.003555*
10406	Ground squirrel hepatitis virus	Hepadnaviridae	0.093975	0.003475*
10372	Human herpesvirus 7	Herpesviridae	0.027847	0.115068

All virus profiles for which a score could be calculated (see Materials and methods) are shown sorted by similarity score. *Statistically significant probabilities (*P *< 0.01).

**Table 4:** Example 1: hepatitis microarray - oligonucleotides contributing to hepatitis B virus profile prediction

Oligonucleotide	Parental virus genome	Virus family	Raw intensity	Raw energy
21326584_16	Hepatitis B virus	Hepadnaviridae	403	102.9
9628700_11_rc	Hepatitis B virus	Hepadnaviridae	316	102.9
9634216_16	Orangutan hepadnavirus	Hepadnaviridae	357	96.6
21326584_25	Hepatitis B virus	Hepadnaviridae	262	109.6
9634216_11_rc	Orangutan hepadnavirus	Hepadnaviridae	308	99.1
9634216_11	Orangutan hepadnavirus	Hepadnaviridae	288	99.1
9630370_16	Woolly monkey hepatitis B virus	Hepadnaviridae	464	72.2
9628700_20_rc	Hepatitis B virus	Hepadnaviridae	160	120
21326584_9	Hepatitis B virus	Hepadnaviridae	175	104.7
9628700_4	Hepatitis B virus	Hepadnaviridae	153	104.7

Ten oligonucleotides contributing most to the hepatitis B virus similarity score are shown sorted by their relative contribution (product of normalized intensity and normalized energy values).

**Table 5:** Example 2 - FluA, RSV double infection

Taxonomy ID	Virus profile	Virus family	Similarity score	Probability
11320	Influenza A virus	Orthomyxoviridae	0.504133	0.000000*
183764	Influenza A virus	Orthomyxoviridae	0.486601	0.000000*
130760	Influenza A virus	Orthomyxoviridae	0.105047	0.000151*
11250	Human respiratory syncytial virus	Paramyxoviridae	0.033523	0.007895*
12814	Respiratory syncytial virus	Paramyxoviridae	0.022144	0.007512*
11246	Bovine respiratory Syncytial virus	Paramyxoviridae	0.009983	0.029254
162145	Human metapneumovirus	Paramyxoviridae	0.001604	0.467995

All virus profiles for which a score could be calculated (see Materials and methods) are shown sorted by similarity score. *Statistically significant probabilities (*P *< 0.01).

**Table 6:** Example 3: SARS microarray

Taxonomy ID	Virus profile	Virus family	Similarity score	Probability
Iteration 1				
227859	SARS coronavirus	Coronaviridae	0.415354	0.000001*
219688	Mink astrovirus	Astroviridae	0.335302	0.000000*
70793	Turkey astrovirus	Astroviridae	0.217455	0.000000*
11120	Avian infectious bronchitis virus	Coronaviridae	0.175788	0.000004*
70794	Ovine astrovirus	Astroviridae	0.153207	0.000031*
107033	Avian nephritis virus	Astroviridae	0.057325	0.000020*
47001	Equine rhinitis B virus	Picornaviridae	0.048009	0.000054*
12702	Human astrovirus	Astroviridae	0.044928	0.002118*
11852	Simian type D virus 1	Retroviridae	0.034479	0.016202
31631	Human coronavirus OC43	Coronaviridae	0.029834	0.002178
Iteration 2				
11852	Simian type D virus 1	Retroviridae	0.053705	0.007108*
39068	Mason-Pfizer monkey virus	Retroviridae	0.031347	0.026931
10359	Human herpesvirus 5	Herpesviridae	0.024634	0.167435
147712	Human rhinovirus B	Picornaviridae	0.022551	0.048232
208177	Tomato leaf curl Vietnam virus	Geminiviridae	0.022090	0.149573
85752	Tomato yellow leaf curl Thailand virus	Geminiviridae	0.021844	0.080110
223334	Tobacco leaf curl Kochi virus	Geminiviridae	0.021469	0.108687
188763	Chimpanzee cytomegalovirus	Herpesviridae	0.021088	0.132918
32610	Tomato geminivirus	Geminiviridae	0.021055	0.081960
83839	Pepper leaf curl virus	Geminiviridae	0.020882	0.082562

For each iteration, ten profiles with highest similarity scores are shown sorted by score. *Statistically significant probabilities (*P *< 0.01). SARS, severe acute respiratory syndrome.
